# 221 K Local Photothermal Heating in a Si Plasmonic Waveguide Loaded with a Co Thin Film

**DOI:** 10.3390/s21196634

**Published:** 2021-10-06

**Authors:** Nana Ota, Tomohiro Miyauchi, Hiromasa Shimizu

**Affiliations:** 1 Department of Electrical and Electronic Engineering, Tokyo University of Agriculture and Technology, Koganei, Tokyo 184-8588, Japan; s218114t@st.go.tuat.ac.jp; 2 Department of Industrial Technology and Innovation, Tokyo University of Agriculture and Technology, Koganei, Tokyo 184-8588, Japan; miyatuat@gmail.com; 3 Department of Applied Physics and Chemical Engineering, Tokyo University of Agriculture and Technology, Koganei, Tokyo 184-8588, Japan

**Keywords:** optical waveguide, plasmonic waveguide, local heating, photonic integrated circuit, optical sensors, surface plasmon polariton, photothermal effect

## Abstract

Photothermal heaters are important devices for optical switches and memories based on the thermo-optic/magneto-optic effect and phase change materials. We demonstrated photothermal heating in Si plasmonic waveguides loaded with Co thin films by measuring the resistance change upon inputting transverse-magnetic (TM) mode light. Temperature rise is proportional to the light intensity with clear polarization dependence. The photothermal conversion efficiency was estimated at 36 K/mW and maximum temperature rise was estimated at 221 K at steady state upon the inputting 6.3 mW TM mode light for the 400 nm-wide, 8 µm-long and 189 nm-thick Co film deposited on the Si wire waveguide with 129 nm-thick SiO_2_ buffer layer. The method to increase the efficiency is discussed based on the experimental and simulation results considering the thickness of the SiO_2_ buffer layer, Co layer and Si core layer, waveguide width, and wavelength. Local photothermal heaters in this study can be applied to a variety of fields including optical switches/memories without electrical control signals in photonic integrated circuits, on-chip optical sensors, and a lab-on-a-chip in biology, chemistry, and medicine.

## 1. Introduction

Recent development of information and communication technology requires larger and larger bandwidth. Optical interconnects based on photonic integrated circuits (PICs) meet this demand and will play important roles in overcoming the limited bandwidth of electrical circuits. However, there are two problems for a dense integration of optical devices. One problem is the size of optical components. The length of an optical component is limited by the light wavelength, the relatively long length of interaction between light and matter (e.g., the electro-optical (EO), magneto-optical (MO), and thermo-optical (TO) interaction) and the large minimal bending radius of an optical waveguide. Another problem is realization of the optical devices without an electrical control signal in PICs for larger bandwidth. In particular, the realization of optical switches and memories, which can be operated by optical signals, is important in order to overcome the bandwidth of electrical circuits. Optical switches based on phase shifters with the TO effect have been reported with 100 μm-long Si waveguides and current application [1]. It is possible to realize heating in smaller area by combination of strong optical confinement and optical signals, without thermal crosstalk.

It is possible to reduce the size of optical components when an optical waveguide with a strong optical confinement such as a Si nanowire waveguide or a plasmonic waveguide are used [1,2,3,4,5]. The optical confinement in a Si nanowire waveguide is strong, because of a high refractive index contrast between Si and SiO_2_. Surface plasmon polaritons (SPPs) are quasiparticles by collective electron oscillations coupled to electromagnetic waves at the interface between materials having positive and negative permittivity, typically a dielectric and a metal. The use of a plasmonic waveguide benefits for a denser integration. Optical confinement in a plasmonic waveguide is stronger than in a Si nanowire waveguide, because the light is confined by a metal/dielectric interface beyond the diffraction limit. It makes the optical confinement stronger and enables stronger interaction between light and matter [5].

The interaction between light and matter with SPP has been applied to optical refractive index sensors with propagating/localized surface plasmon resonance (SPR) [6], localized surface plasmon with enhanced Raman scattering (SERS) [7], magnetic modulation of SPP and optical isolator application [8], and light-induced local heating with metal nanoparticles [9,10]. Light-induced local heating has been applied for thermophoretic manipulation of DNA with Au nanoparticles [11], resonant light-triggered DNA release from plasmonic nanoparticles [12], and selective light-induced contents release from liposomes [13]. It was reported that the liquids inside the micro- and nanochannels were heated to approximately 41 °C using a laser power of 38 mW [11].

A Si waveguide combined with propagating SPP is one of the choices used in order to realize a lot of functions such as light-guiding, optical modulating [14], polarization handling, heating, and optical sensing [6], in nanoscale integration. Propagating SPPs combined with a Si waveguide have the advantages of the capability of integrating light sources, detectors, and input/output optical fibers on a single chip in a scheme of PICs.

There have been reports on local heating in Si hybrid nanoplasmonic devices [5,15], where the joule heating by the propagation loss owing to the interaction between light and metal is used. The local heaters are important devices for optical switches based on the TO and MO effect [16], optical memories loaded with phase change materials [17,18,19], and a lab-on-a-chip for biology, chemistry, and medicine [11,12,13]. Heating to 200 °C is required for optical memory applications with phase change materials such as Ge_2_Sb_2_Te_5_ (GST) [17,18,19]. The magneto-optical disk employed MO materials (GdFeCo, and TbFeCo) as recording media, and when the temperature is high enough to reach the Curie temperature of the MO materials (~200 °C), their magnetization direction can be reversed and stored after cooling [16]. Heat-assisted magnetic recording (HAMR) uses heat from a laser beam confined below the diffraction limit (<50 nm-wide), in order to write the media of large perpendicular magnetic anisotropy at near 450 °C [20].

In a previous study of Si hybrid nanoplasmonic devices, a heating efficiency of 214 K/mW and heating with 340 K for a power consumption of 1.6 mW was theoretically reported in a 10 μm-long Au thin film loaded on 100 nm-wide Si nanowire waveguide [5]. A propagation loss of 0.35 dB/μm and a heating efficiency of 49 K/mW were experimentally estimated in a Si nanowire ring waveguide resonator partially loaded with a 20 nm-thick and 5 μm-long Ti film, by measuring the shift of the resonance wavelength for different optical intensities [15]. The rise time upon the light input is faster than the typical time span of sweeping the wavelength for measuring the resonance characteristics, such that it is difficult to estimate the amount of the temperature rise and investigate the influence of the length, width, and thickness of the metal thin film on the heating characteristics. It is important to measure the temperature rise and rise time more precisely for various applications.

Measurement of the resistance of the metal thin film deposited on top of the Si waveguide is one of the simpler methods used to estimate the temperature rise and local photothermal heating. In this paper, we report the fabrication of Si plasmonic waveguides loaded with Co thin films, whose metal resistance changes are measured by a pair of electrode pads. Estimated temperature rise is proportional to the light intensity with clear polarization dependence, and the thermal conversion efficiency was estimated to 36 K/mW for transverse magnetic (TM) mode light. Maximum temperature rise was estimated to 221 K upon the inputting 6.3 mW TM mode light at steady state. The influence of the width of the Co thin film deposited on the Si waveguides was investigated.

## 2. Design of the Si Plasmonic Waveguide Heater

**Figure 1:**
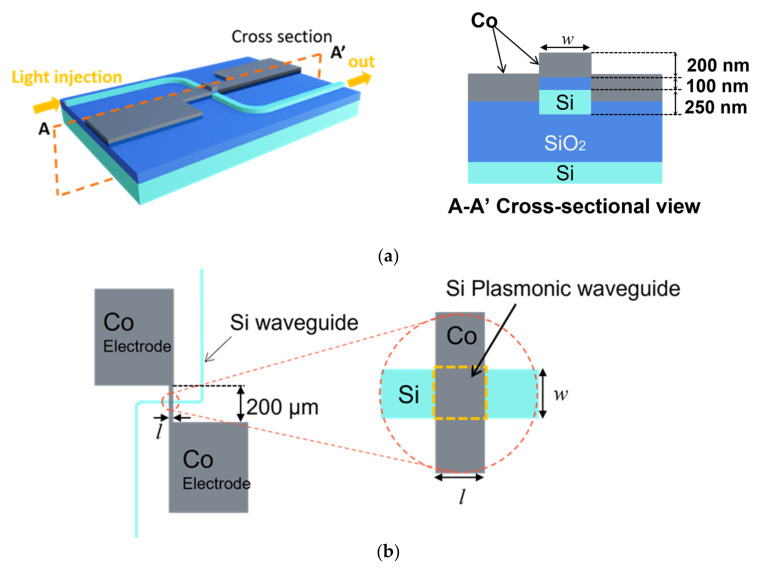
Schematic images of a Si plasmonic waveguide heater with the width of *w*, and length of *l*. (**a**) Entire image and cross-sectional image of the Si plasmonic waveguide with optical input waveguide and a pair of electrodes. (**b**) Dimension of the Si plasmonic waveguide heater loaded with a Co thin film, connected to a pair of electrode pads.

We show the design of Si plasmonic waveguide heaters loaded with Co thin films. Transition metals are more appropriate than noble metals from the viewpoints of local heating by the propagation loss of SPP. Resistor material such as Ti, W or Co has an upper limit of temperature rise and is appropriate from the viewpoint of a long-term reliability. One of the important factors determining the propagation loss is the imaginary part of the relative dielectric constant. We selected Co as having relative permittivity, −38.0 + 50.7i at a wavelength of 1550 nm, based on [21]. Co is less oxidized than Fe, and ferromagnetic metal whose Curie temperature is 1380 K. The temperature rise at the interface of the Co layer and the Si wire waveguide can be measured based on the temperature coefficient of the resistivity of Co (7.0 × 10^−3^ 1/K) [22]. Figure 1 shows the schematic image of the Si plasmonic waveguide heater with a pair of electrode pads for measuring the resistance. The width *w* of the Si wire waveguide and Co film on top of the waveguide were set to 400, and 1000 nm in order to investigate the influence of the width and optical confinement at the metal/dielectric interface on the heating efficiency. An S-bend waveguide with a bending radius of 50 μm is used as the input and output waveguide in order to discriminate the output light from the stray light by coupling the light by a pair of lensed fibers. The height *h* of the Si wire waveguide is 250 nm. A 200 nm-thick Co thin film is used as the metal with 100 nm-thick SiO_2_ layer on the Si wire waveguide in order to realize the plasmonic waveguide having a propagation loss of 1.0 dB/µm.

When all of the propagation loss is converted to Joule loss, the absorbed surface energy density is as large as 2.5 × 10^7^ W/m^2^ for input light intensity of 1 mW and *w* = 400 nm, which is 2.5 × 10^4^ times larger than that of the power density of sunlight at Air Mass 1.5. The length *l* of the Co film on top of the Si wire waveguide was set at 8 μm.

## 3. Fabrication of the Si Plasmonic Waveguide Heaters with Co Electrodes

**Figure 2:**
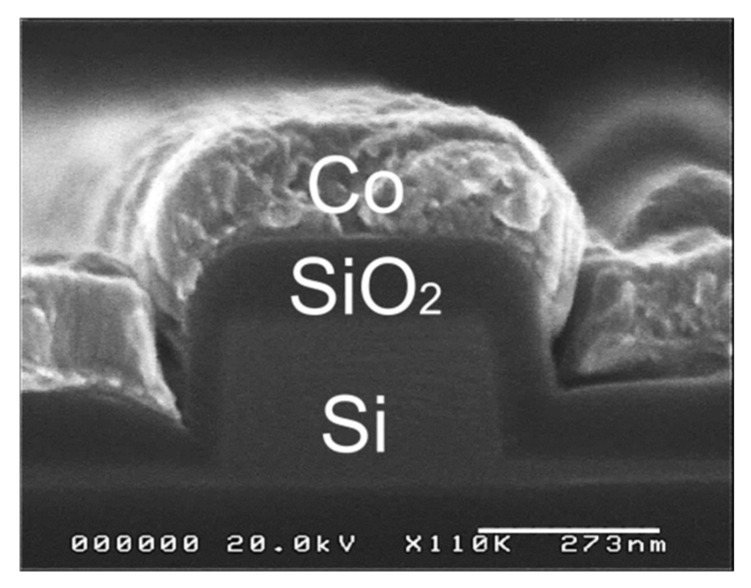
A cross sectional SEM image of Si plasmonic waveguide.

**Figure 3:**
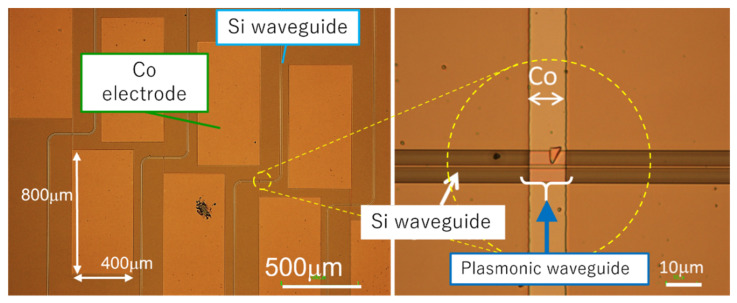
Optical microscope images of the Si plasmonic waveguide heater with electrode pads.

The designed waveguide heaters were fabricated on an SOI substrate by electron-beam (EB) writing and reactive ion etching. The SiO_2_ buffer layer and Co layer were deposited on the Si wire waveguides by sputtering and EB deposition with a lift-off process. Figure 2 shows the cross-sectional image taken by a scanning electron microscope (SEM). The thickness of the deposited SiO_2_ buffer layer was 129 nm, which is slightly larger than the designed thickness (100 nm). The thickness of the Co thin film was 189 nm, which is slightly smaller than the designed thickness (200 nm). Since the Co layer is also used as the electrical resistance, the Co film was formed at the both sides of the Si plasmonic waveguide and was connected with a pair of electrode pads (0.40 mm × 0.80 mm) with a distance of 200 μm, as an optical microscope image shows in Figure 3. The distance between the both facets including the input and output waveguides is 3.5 mm.

## 4. Measurement of the Resistance Change and Rise Time for Characterization of the Photothermal Heating Efficiency

**Figure 4:**
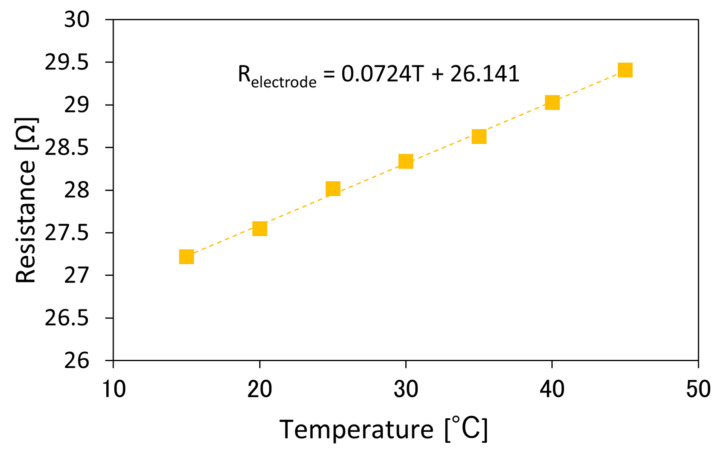
Temperature dependence of the resistance of the Co layer loaded on the Si waveguide (*w* = 400 nm, and *l* = 8 µm) without light injection.

First, we measured the temperature dependence of the resistance of the deposited Co thin film by two probes method with a digital multimeter as a reference of the local photothermal heating efficiency. Temperature of the whole waveguide was set between 15 and 45 °C. The temperature of the whole waveguide was controlled by a thermoelectric controller. Figure 4 shows the temperature dependence of the resistance of the Co layer loaded on the Si waveguide (*w* = 400 nm, and *l* = 8 μm) without light injection. The temperature coefficient of the resistance was 7.24 × 10^−2^ Ω/K, which is slightly larger than the estimated value (5.2 × 10^−2^ Ω/K) based on the temperature coefficient of the resistivity of Co [22], distance between the electrode pads (*l*_Co_ = 200 μm), width (*w* = 400 nm), and thickness (189 nm) of the deposited Co thin film. One of the reasons for the deviation between the experimental and estimated values is that the surface of the deposited Co thin film is slightly oxidized and the resistivity is larger than the estimated value based on the ref. [21].

Then, we measured the resistance change of the Co layer upon the light injection and estimated the temperature rise. TM mode light with a wavelength of 1550 nm was coupled with a lens fiber for exciting SPP at the interface of Co and SiO_2_ layer. The temperature of the device before the measurement was 22.1 °C (room temperature). No temperature control was applied to the device during the measurement. The resistance between the electrodes was measured with a digital multimeter, for intensity of 0 to 8 dBm (1 to 6.3 mW). Transverse electric (TE) mode light was also input for reference. The resistance before and after the light injection was recorded.

**Figure 5:**
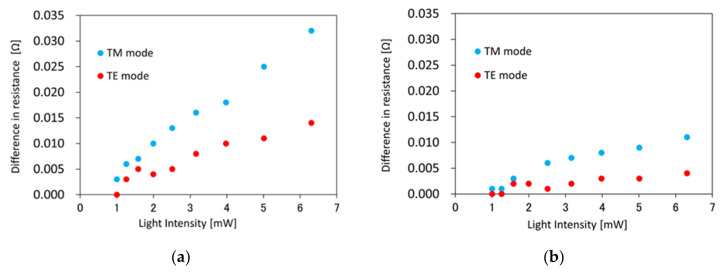
Difference in resistance Δ*R*_light_ of the Co layer with the Si waveguides having the waveguide width *w* of (**a**) 400, and (**b**) 1000 nm, upon TM and TE mode light injection at steady state.

Figure 5 shows the resistance rise Δ*R*_light_ at steady state of the Co layer with the Si waveguides having the waveguide width *w* of (a) 400, and (b) 1000 nm, for TM and TE mode light. The resistance rise was larger with TM mode light than with TE mode light, and larger with narrower waveguide (*w* = 400 nm) than wider waveguide (*w* = 1000 nm). The resistance changes before and after the input of TM mode light is proportional to the input light intensity, and the efficiencies are 5.2 × 10^−3^ Ω/mW for *w* = 400 nm, and 1.9 × 10^−3^ Ω/mW for *w* = 1000 nm. The maximum resistance change Δ*R*_max_ was 0.048 Ω for *w* = 400 nm, and 1.6 s after injection of 6.3 mW TM mode light, whereas the resistance change Δ*R*_steady_ was 0.032 Ω for the same condition at steady state. When we assume that the resistance changed locally on the top of the waveguide (the area of *w* × *l*) by light injection, and unchanged outside the waveguide, the local temperature rise Δ*T*_local_ for the Si plasmonic waveguide can be estimated from the temperature dependence of the resistance of the whole electrode (ΔRCoΔT = 7.24 × 10^−2^ Ω/K) of Figure 4. Since the temperature dependence of the whole electrode is expressed as $\frac{\Delta R_{\mathrm{Co}}}{\Delta T}=\frac{\Delta \rho }{\Delta T}\frac{l_{\mathrm{Co}}}{ld}$, the local temperature dependence of the resistance of the Co thin film on top of the waveguide is expressed as $\frac{\Delta R_{\mathrm{local}}}{\Delta T_{\mathrm{local}}}=\frac{\Delta \rho }{\Delta T_{\mathrm{local}}}\frac{w}{ld}$ and the temperature coefficient of the resistivity ($\frac{\Delta \rho }{\Delta T},\;\frac{\Delta \rho }{\Delta T_{\mathrm{local}}}$) is constant, and can be derived by following Equation (1).

(1) ΔTlocal=1ΔRCoΔT×ΔRlight×lCo w

**Figure 6:**
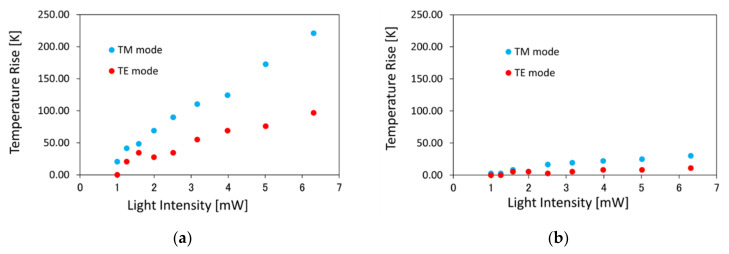
Estimated local temperature rise for TM and TE mode light with the Si waveguides having the waveguide width *w* = (**a**) 400 nm, and (**b**) 1000 nm.

Figure 6 shows the estimated local temperature rise for TM and TE mode light with the Si waveguides having the waveguide width *w* = (a) 400 nm and (b) 1000 nm. Estimated temperature rise was 221 K for 6.3 mW TM mode light at steady state (Δ*R*_steady_ = 0.032 Ω). Please note that temperature of the device before the measurement was 22.1 °C, and the device is heated up to 22.1 + 221 = 243.1 °C. The heating efficiency was estimated to 36 K/mW and 16 K/mW for TM and TE mode light. Δ*R*_max_ = 0.048 Ω corresponds to temperature rise of 331 K.

**Figure 7:**
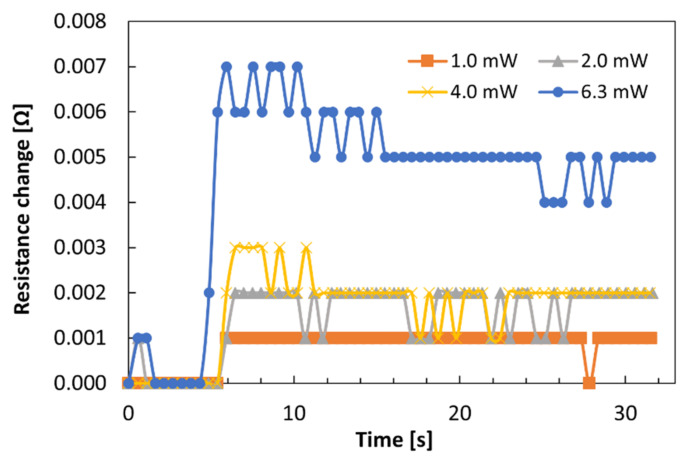
Transition of the measured resistance change after light injection of 1.0, 2.0, 4.0, and 6.3 mW for TM mode light with the Si waveguides with 200 nm-thick SiO_2_ buffer layer. Input light was injected at *t* = 4~5.5 s.

In order to measure the rise time as well as temperature rise, we measured the transition of the resistance after light injection. We fabricated another Si plasmonic waveguide heater loaded with a Co thin film. The waveguide width is 400 nm, and thickness of the SiO_2_ buffer layer is 194 nm, which is larger than that of the device measured in Figure 2, Figure 3, Figure 4, Figure 5 and Figure 6. The length *l* of the Co film on top of the Si wire waveguide is 10 μm. The distance between the pair of electrode pads is 50 μm, which is smaller than that of Figure 2, Figure 3, Figure 4, Figure 5 and Figure 6 (200 μm). The temperature dependence of the resistance of the whole electrode ΔRCoΔT is 2.01 × 10^−2^ Ω/K. We measured the transition of the resistance change by a two-probe method with a digital multimeter. Figure 7 shows the transition of the resistance change after light injection with the intensity of 0 to 8 dBm (1 to 6.3 mW) and time interval of 0.5 s. The maximum temperature change of 0.007 Ω was detected for intensity of 6.3 mW and 1 s after light injection. The transition time (1 s) is the same for the all light intensities. Measurement with shorter time interval is necessary for more precise analysis of relationship between the rise time and light intensity, which is one of the future issues. The estimated temperature rise is 44 K, which is 1/5 of that of the device described in Figure 6a.

## 5. Discussion

We discuss the validity of Equation (1) for estimation of the temperature rise. The temperature dependences of the resistance of the whole electrode ΔRCoΔT are 7.24 × 10^−2^ Ω/K for *l*_Co_ = 200 μm and *l* = 8 μm and 2.01 × 10^−2^ Ω/K for *l*_Co_ = 50 μm and *l* = 10 μm. ΔRCoΔT is 3.6 time larger for *l*_Co_ = 200 μm and *l* = 8 μm, compared with that for *l*_Co_ = 50 μm and *l* = 10 μm. Since ΔRCoΔT is proportional to the distance between the pair of electrode pads *l*_Co_ and inversely proportional to the area of the cross section (*w* x *l*), ΔRCoΔT is five times larger for *l*_Co_ = 200 μm and *l* = 8 μm, compared with that for *l*_Co_ = 50 μm and *l* = 10 μm. The relationship of ΔRCoΔT between the fabricated two samples (3.6 time) can be explained by influences of *l*_Co_ and *l* on ΔRCoΔT, which provides the validity of Equation (1).

**Figure 8:**
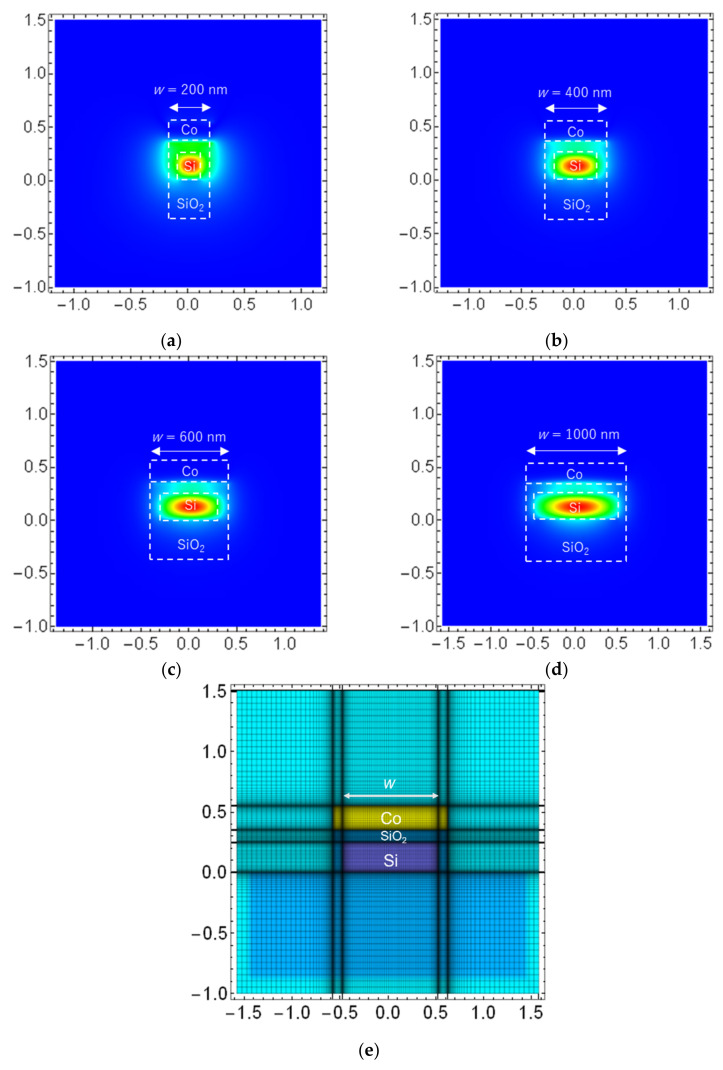
Calculated profile of the horizontal component of the magnetic field (absolute value $\left|H_{y}\right|$) for TM-like mode light with the Si waveguides for waveguide width (**a**) *w* = 200 nm, (**b**) 400 nm, (**c**) 600 nm, and (**d**) 1000 nm. (**e**) Definition of the mesh for calculation of the mode profile. The interfaces between the Co, SiO_2_, and Si layers are shown by white dotted lines.

Furthermore, we discuss the influence of the polarization and waveguide width *w* on the resistance change of the Co layer with the Si waveguides. The resistance change upon inputting the TM mode light is 2.7 times larger than that with TE mode light for *w* = 400 nm. Since the propagation loss is larger for TM mode light, owing to excitation of the SPPs at the Co/SiO_2_/Si interface with TM mode light, the polarization dependence of the resistance change is clear evidence of local photothermal heating at the Co-loaded Si plasmonic waveguides. Δ*R*_light_ at steady state upon injecting 6.3 mW TM mode light is 0.032 Ω for *w* = 400 nm, which is 2.9 times larger than that for *w* = 1000 nm. The reason for larger Δ*R*_light_ for *w* = 400 nm is that the propagating light in the Si plasmonic waveguide is confined to the narrower region along the width direction. Figure 8 shows the profile of the horizontal component of the magnetic field (absolute value $\left|H_{y}\right|$) for TM-like mode light with the Si waveguides having the waveguide width (a) *w* = 200 nm, (b) 400 nm, (c) 600 nm, and (d) 1000 nm, calculated by the finite-difference frequency-domain (FDFD) method [23]. Figure 8e shows the definition of the mesh for calculation of the mode profile and effective refractive index. From Figure 8, the field distribution at the SiO_2_/Co interface becomes more uniform with decreasing the waveguide width *w*, owing to the horizontal optical confinement. The border line between uniform and non-uniform field distributions is between *w* = 200 and 400 nm. The difference of the field distribution (uniformity) brings the difference of the heating property and resistance rise. It is possible to improve the heating efficiency by reducing the width of the Si waveguide (*w* = 200 nm).

Estimated temperature rise of 331 K with Δ*R*_max_ = 0.048 Ω for *w* = 400 nm was determined with sampling time interval (1 s) of the digital multimeter used in this study, so that smaller temperature rise is expected to be measured with a higher time resolution. Here, please note that the estimated temperature rise gives the average temperature rise of the Co thin film on top of the waveguide by photothermal heating, because the Co thin film is not a guided layer, and the temperature is distributed across the waveguide cross section including the Co thin film, SiO_2_ buffer layer, and Si core layer.

In order to reduce the required light intensity, it is important to realize uniform field distribution by narrowing the waveguide and increasing the propagation loss by decreasing the Co layer thickness, SiO_2_ buffer layer thickness, and Si core layer thickness. Influence of the Co layer thickness and SiO_2_ buffer layer thickness on the propagation loss for fixed Si core layer thickness of 250 nm and wavelength of 1550 nm, are summarized in Table 1. The propagation loss from the Co layer increased with decreasing the thickness by exciting SPP at lower and upper interfaces of the Co layer. Thinning the Co layer is effective for decreasing the coupling loss between the Si plasmonic waveguide and Si wire waveguides, owing to smaller difference of the optical mode profiles, so that equivalent input power can be enhanced with the same input power from the input Si waveguide, which will contribute to a decrease of the required power level in the waveguide. Decreasing the SiO_2_ buffer layer thickness is effective for increasing the propagation loss. By changing the Co layer thickness from 200 to 50 nm, and SiO_2_ buffer layer thickness from 100 to 10 nm, the propagation loss can be increased to 2.9 dB/μm, meaning that the required light intensity is 2.9 times smaller than that of the device reported in Section 4. By decreasing the Si core layer thickness, it is possible to suppress the dielectric mode and enhance the field intensity at the Co/SiO_2_ interface as hybrid mode, leading to a larger propagation loss and photothermal efficiency. The influence of the Si core layer’s thickness on propagation loss is summarized in Table 2. With increasing the wavelength, size of the mode profile and field intensity at the Co/SiO_2_ interface increase, leading to larger propagation loss and photothermal efficiency. The influence of the wavelength on propagation loss is summarized in Table 3. Narrowing the waveguide width gives smaller anisotropy and difference of the effective refractive index and mode profile between TM and TE mode light, which leads to the possibility of being less sensitive to the polarization of the input signals from the standpoint of practical optical functional device applications. By thinning the Co layer thickness, narrowing the waveguide width, decreasing the SiO_2_ buffer layer thickness and Si core layer thickness, enhancement of photothermal heating with shorter interaction length between the light and matter, and denser integration of Si plasmonic waveguide heaters can be expected for optical memory applications with phase change materials and HAMR.

## 6. Conclusions

We have reported the design, fabrication, and characterization of Co-loaded Si plasmonic waveguide heaters by measuring the resistance change. The resistance change is proportional to the input power and dependent on polarization, and inversely proportional to the waveguide width. The resistance change is measured with a transition time shorter than 1 s. Estimated local temperature rise was 221 K for 6.3 mW TM mode light for a 400 nm-width, 189 nm-thick, and 8 μm-long waveguide. Photothermal heating efficiency was calculated as 36 K/mW. The local heating characteristics obtained in the Si plasmonic waveguides in this study can be applied for a wide range of optical device applications, including phase-change and MO materials for non-volatile optical memories, on-chip optical sensors, and a lab-on-a-chip in biology, chemistry, and medicine. Further improving of the waveguide structure measured with higher time resolution could lead to higher temperature rise, and will pave the way to a wide variety of applications from on-chip non-volatile memories to lab-on-a-chip.

## Figures and Tables

**Table 1:** Calculated propagation loss in a unit of dB/μm with combinations of different SiO_2_ buffer layer thickness *d*_SiO2_ and Co layer thickness *d*_Co_ for fixed Si core layer thickness of 250 nm and wavelength of 1550 nm.

	Propagation Loss [dB/μm]			
	*d*_Co_ = 50 nm	*d*_Co_ = 100 nm	*d*_Co_ = 150 nm	*d*_Co_ = 200 nm
*d*_SiO2_ = 50 nm	2.90	2.12	2.02	2.00
*d*_SiO2_ = 100 nm	1.55	1.08	1.02	1.00
*d*_SiO2_ = 150 nm	0.92	0.63	0.59	0.57
*d*_SiO2_ = 200 nm	0.55	0.37	0.35	0.34

**Table 2:** Calculated propagation loss with Si core layer thicknesses of 200 and 250 nm, for fixed SiO_2_ buffer layer thickness of 100 nm, Co layer thickness of 200 nm, and wavelength of 1550 nm.

Si core Layer Thickness [nm]	Propagation Loss [dB/μm]
200	1.67
250	0.93

**Table 3:** Calculated propagation loss with different wavelengths of 1350, 1450, and 1550 nm, for fixed Si core layer thickness of 250 nm, SiO_2_ buffer layer thickness of 100 nm, and Co layer thickness of 200 nm.

Optical Wavelength [nm]	Propagation Loss [dB/μm]
1350	0.43
1450	0.67
1550	0.93

## Data Availability

The data presented in this study are available in insert article.
